# Test–retest reliability of laser evoked pain perception and fMRI BOLD responses

**DOI:** 10.1038/s41598-020-79196-z

**Published:** 2021-01-14

**Authors:** Yanzhi Bi, Xin Hou, Jiahui Zhong, Li Hu

**Affiliations:** 1 CAS Key Laboratory of Mental Health, Institute of Psychology, Chinese Academy of Sciences, Beijing, 100101 China; 2 Department of Psychology, University of Chinese Academy of Sciences, Beijing, 100101 China; 3 Research Center of Brain and Cognitive Neuroscience, Liaoning Normal University, Dalian, 116029 China

**Keywords:** Perception, Brain

## Abstract

Pain perception is a subjective experience and highly variable across time. Brain responses evoked by nociceptive stimuli are highly associated with pain perception and also showed considerable variability. To date, the test–retest reliability of laser-evoked pain perception and its associated brain responses across sessions remain unclear. Here, an experiment with a within-subject repeated-measures design was performed in 22 healthy volunteers. Radiant-heat laser stimuli were delivered on subjects’ left-hand dorsum in two sessions separated by 1–5 days. We observed that laser-evoked pain perception was significantly declined across sessions, coupled with decreased brain responses in the bilateral primary somatosensory cortex (S1), right primary motor cortex, supplementary motor area, and middle cingulate cortex. Intraclass correlation coefficients between the two sessions showed “fair” to “moderate” test–retest reliability for pain perception and brain responses. Additionally, we observed lower resting-state brain activity in the right S1 and lower resting-state functional connectivity between right S1 and dorsolateral prefrontal cortex in the second session than the first session. Altogether, being possibly influenced by changes of baseline mental state, laser-evoked pain perception and brain responses showed considerable across-session variability. This phenomenon should be considered when designing experiments for laboratory studies and evaluating pain abnormalities in clinical practice.

## Introduction

Blood oxygenation level-dependent functional magnetic resonance imaging (BOLD fMRI) can measure human brain activity objectively and enable a deep understanding of neural processing mechanisms. With this technique, the function of pain pathway structures within the central nervous system in states of acute or chronic pain^1–6^, during therapeutic and psychological pain interventions^3,7–9^ has been extensively investigated. Recently, accumulating studies suggested that fMRI could be used as a technique to elucidate objective biomarkers for the diagnosis of pain^10,11^ as the magnitude of neural responses and self-report pain ratings are highly correlated in many situations^12^.

However, pain perception is a very subjective experience, and self-report of pain perception reflects a complex mix of physiological and psychological processes, including nociception, emotion, decision making, self-awareness, social cognition, and communicative tendencies^13^. It is important to note that, even in the same condition, pain experience is not static over time within one individual, and pain perception can fluctuate from time to time^14^. Brief painful heat pulses, as generated by infrared laser stimulator, have been adopted as a selective way to activate nociceptive Aδ- and C-fiber afferents and have been widely used to assess the function of the nociceptive system in humans^15,16^. It is well known that radiant-heat laser stimuli could induce pure pain (without tactile sensation) and robust BOLD activation and deactivation in multiple brain structures, which are responsible for the processing of the sensory, cognitive, and affective components of pain^17^. However, few studies have examined whether the pain perception evoked by radiant-heat laser stimuli and the associated BOLD responses remain stable over time. In other words, the test–retest reliability of laser-evoked pain perception and BOLD responses remains unassessed.

To address this issue, an experiment with a within-subject repeated-measures design was performed in 22 healthy volunteers. Radiant-heat laser stimuli were delivered on the dorsum of subjects’ left hand to evoke pain perception and BOLD responses in two different sessions, which were separated by 1–5 days. With both behavioral and BOLD fMRI data (task fMRI and resting-state fMRI), we assessed (1) whether laser-evoked pain perception (i.e., pain threshold and pain ratings) and BOLD responses were significantly different between the two sessions; (2) the test–retest reliability of laser-evoked pain perception and BOLD responses in healthy subjects; (3) the possible influence of the changes of resting-state brain activity on the modulation of laser-evoked pain perception and BOLD responses.

## Results

### Behavioral results

Twenty-two healthy, right-handed, and pain-free male volunteers (mean age ± standard deviation: 26.73 ± 3.37 years, mean education years ± standard deviation: 17.41 ± 2.04 years) were recruited in this study. Each subject participated in two sessions (session 1: T1; session 2: T2) separated by 1–5 days (1.82 ± 1.12 days). All subjects completed the two experiment sessions. As compared with T1 session, the pain threshold was significantly larger (T1: 2.68 ± 0.33, T2: 2.84 ± 0.43, *p* = 0.04; Table 1), and pain ratings to laser stimuli were significantly lower (T1: 5.12 ± 1.14, T2: 4.59 ± 0.95, *p* = 0.004; Table 1) in T2 session. The skin temperature (T1: 31.63 ± 0.97, T2: 31.46 ± 0.92, *p* = 0.334; Table 1) and state anxiety (State Anxiety Inventory, SAI; T1: 32.86 ± 8.64, T2: 32.64 ± 10.43, *p* = 0.82; Table 1) were not significantly different between the two sessions, which excluded the possible influences of skin temperature and state anxiety on the changes of pain perception between the two sessions.

**Table 1:** Demographics and behavioral results.

Variables	T1		T2		*p* value
	Mean	SD	Mean	SD	
Age, in year	26.73	3.37			
Education, in year	17.41	2.04			
BDI	2.73	4.1			
SAI	32.86	8.64	32.64	10.43	0.82
TAI	38.23	8.54			
Skin temperature, in °C	31.63	0.97	31.46	0.92	0.334
Pain-related					
PSQ	4.42	1.30			
Pain threshold, in J	2.68	0.33	2.84	0.43	0.04
Pain rating	5.12	1.14	4.59	0.95	0.004

*BDI* Beck Depression Inventory, *SAI* State Anxiety Inventory, *TAI* Trait Anxiety Inventory, *PSQ* pain sensitivity questionnaire, *SD* standard deviation.

### Brain responses to nociceptive stimuli

**Figure 1:**
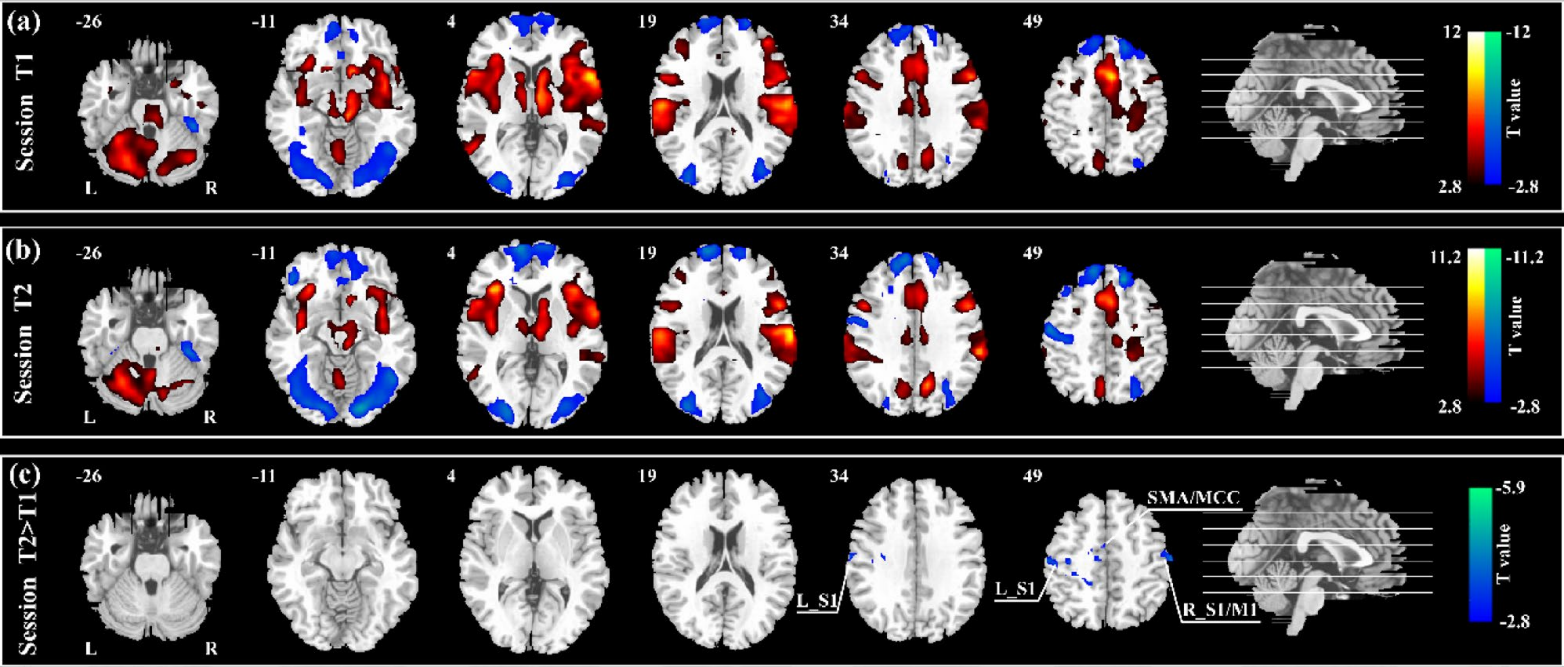
Brain responses to nociceptive laser stimuli. For both T1 session (**a**) and T2 session (**b**), nociceptive stimuli elicited significant brain responses in the periaqueductal gray, thalamus, primary somatosensory cortex (S1), secondary somatosensory cortex, insula, and anterior cingulate cortex. (**c**) Laser-evoked brain responses were significantly smaller in bilateral S1, right primary motor cortex (M1), supplementary motor area (SMA), and middle cingulate cortex (MCC) during T2 session than T1 session. *L* left hemisphere; *R* right hemisphere. The resulting t-statistic maps were thresholded at *p* < 0.005, cluster-corrected at family wise error of *p* < 0.05. The images were created by using the MRIcron software (v1.0.20190902, https://www.nitrc.org/projects/mricron).

For both sessions, nociceptive stimuli elicited significant activations in a wide array of subcortical and cortical brain regions, including the periaqueductal gray, thalamus, primary (S1) and secondary (S2) somatosensory cortices, insula, and the anterior cingulate cortex (ACC) (voxel-wise *p* < 0.005 and cluster-corrected at family wise error (FWE) of *p* < 0.05; Fig. 1a, b). These brain activation patterns strongly resemble the findings previously described^18–21^. The paired-sample t-test revealed that the magnitudes of brain activations were significantly smaller in bilateral S1, primary motor cortex (M1), supplementary motor area (SMA), and middle cingulate cortex (MCC) in T2 session than in T1 session (voxel-wise *p* < 0.005 and cluster-corrected at FWE of *p* < 0.05; Fig. 1c). These results showed that the decrease in pain ratings was accompanied by a reduced BOLD response in brain regions responsible for nociceptive information processing.

### Test–retest reliability

For pain threshold, the intraclass correlation coefficient (ICC) between the two sessions showed fair-reliability (ICC = 0.56; Table 2), while the ICC for pain ratings to nociceptive laser stimuli demonstrated moderate-reliability (ICC = 0.657; Table 2). For both mean BOLD responses (the clusters showing significant differences between the two sessions) and mean region of interest (ROI) BOLD responses (ROIs in the “Pain Matrix”), ICCs also showed fair-to-moderate test–retest reliability (Table 2).

**Table 2:** Intraclass correlation coefficients of pain perception and laser-evoked brain responses between the two sessions.

	ICC	F value	*p* value	CI lower	CI upper
Pain perception					
Pain threshold	0.56	3.986	0.001	0.198	0.789
Pain rating	0.657	6.445	< 0.001	0.233	0.854
Mean BOLD responses					
Left S1	.698	9.922	< 0.001	.091	.893
Right S1/M1	.564	5.606	< 0.001	.030	.820
SMA/MCC	.685	8.901	< 0.001	.113	.884
Mean ROI BOLD responses					
Left anterior insula	.549	3.395	.004	.177	.784
Right anterior insula	.448	2.552	.019	.030	.729
Left posterior insula	.574	3.636	.002	.211	.798
Right posterior insula	.487	2.900	.009	.099	.748
Left ACC	.696	5.621	.000	.404	.860
Right ACC	.682	5.427	.000	.384	.853
Left thalamus	.434	2.510	.020	.026	.718
Right thalamus	.454	2.683	.014	.060	.728
Left S1	.661	6.689	.000	.224	.858
Right S1	.660	6.488	.000	.240	.856
Left S2	.684	5.529	.000	.387	.854
Right S2	.751	7.329	.000	.495	.888

The intraclass correlation coefficients (ICCs) were calculated using the single-rating, absolute-agreement, 2-way mixed model. 95% confidence interval (CI) is the interval between CI lower and CI upper. ROI, region of interest; S1, primary somatosensory cortex; S2, secondary somatosensory cortex; M1, primary motor cortex; SMA, supplementary motor area; MCC, middle cingulate cortex; ACC, anterior cingulate cortex. Bonferroni correction, *p* < 0.025 for pain perception analyses and *p* < 0.05/15 for brain response analyses.

### Spontaneous brain activity and resting-state functional connectivity

**Figure 2:**
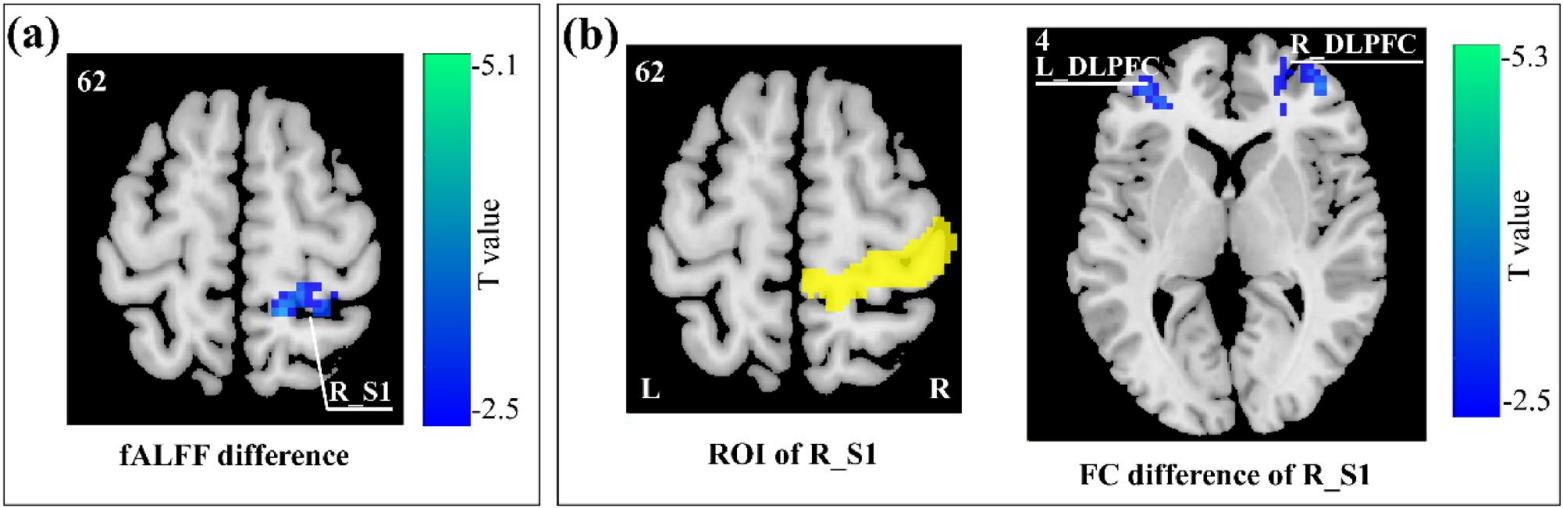
Differences in spontaneous brain activity and resting-state functional connectivity between the two sessions. (**a**) Decreased spontaneous brain activity (fALFF) of the right S1 was observed during T2 session as compared with T1 session. (**b**) When the right S1 was selected as the ROI, the right S1 showed decreased resting-state functional connectivity with the bilateral dorsolateral prefrontal cortex (DLPFC) during T2 session as compared with T1 session. *L* left hemisphere, *R* right hemisphere. The resulting t-statistic maps were corrected for multiple comparisons using the gaussian random field (GRF) theory with a voxel z value > 2.3 and cluster *p* value < 0.05 (two-tailed correction). The images were created by using the Data Processing & Analysis for (Resting-State) Brain Imaging (DPABI) software (DPABI_V5.0_201001, http://rfmri.org/dpabi)^59^.

Decreased spontaneous brain activity (fractional amplitude of low-frequency fluctuations, fALFF) of the right S1 was observed in T2 session as compared with T1 session (paired-sample t-test, voxel z value > 2.3, cluster significance: *p* < 0.05, gaussian random field (GRF) corrected, two-tailed; Fig. 2a). When selecting the right S1 as the ROI for resting-state functional connectivity analysis, we observed that the right S1 exhibited weaker resting-state functional connectivity with the bilateral dorsolateral prefrontal cortex in T2 session as compared with T1 session (paired-sample t-test, voxel z value > 2.3, cluster significance: *p* < 0.05, GRF corrected, two-tailed; Fig. 2b).

## Discussion

As compared with T1 session, declined laser-evoked pain perception coupled with decreased brain responses in bilateral S1, M1, SMA, and MCC were observed in T2 session that was performed 1–5 days later. Test–retest reliability analysis revealed that the ICCs of both pain perception and brain responses elicited by nociceptive laser stimuli were 0.434–0.751, which represented fair-to-moderate reliability. When evaluating the possible effects of baseline spontaneous brain activity on the changes of laser-evoked pain perception and brain responses, we found decreased spontaneous brain activity in the right S1 and decreased resting-state functional connectivity between the right S1 and bilateral dorsolateral prefrontal cortex in T2 session as compared with T1 session. Altogether, being possibly influenced by the modulation of baseline mental state, laser-evoked pain perception and brain responses showed considerable variability between different experiment sessions. This phenomenon should be considered when designing experiments for laboratory studies and evaluating pain abnormalities in clinical practice.

Recent studies showed that mildly noxious stimuli resulted in lower repeatability for experimental pain perception across different sessions than highly painful stimuli^14,22^ and that ratings of pain perception trended downward across sessions^14,22^. These observations were consistent with our findings that laser-evoked pain perception declined significantly from T1 session to T2 session when laser stimuli with a fixed stimulus intensity of 3.5 J elicited a mild-moderate painful sensation (Table 1). One possible explanation of this observation is the difficulty of the rating task for innocuous or mildly noxious stimuli as compared with highly painful stimuli^14^. In addition, the test–retest reliability of pain perception was fair-to-moderate (i.e., ICC = 0.56–0.657, Table 2). By definition, ICC is the ratio of the between-subject variance to the total variance across repeated measures^23^. Any factors that decrease the between-subject variability or increase the within-subject variability will decrease test–retest reliability. In the present study, the declined pain perception from T1 session to T2 session would contribute to the increase of the within-subject variability, which ultimately resulted in decreased reliability of laser-evoked pain perception.

It should be noted that the results at the behavioral level were fully supported by the findings of laser-evoked brain responses: BOLD responses elicited by nociceptive laser stimuli were significantly decreased in bilateral S1, M1, SMA, and MCC across the two sessions (Fig. 1c). Due to the significant decrease of laser-evoked brain responses, the test–retest reliability was not very high, in the range from 0.434 to 0.751, i.e., “fair” to “moderate” reliability. Since the magnitude of neural responses sampled using fMRI technique is capable of encoding subjective pain ratings in most practical situations^12,24,25^, our finding observed at the neural level suggested the reliability of the results obtained at the behavioral level.

It is well known that pain experience is highly variable and could be influenced by factors at different dimensions (e.g., physical, physiological, psychological, social, and cultural factors), which support a multidimensional framework for the modulation of the pain experience^26^. For instance, being stimulated by the same physical stimuli, pain perception would be different when the psychological state (e.g., anxiety level, the fearful level to the stimuli, and the attention level) of the subjects was changed^3,9,27–29^. In the present study, the psychological state of subjects could be different between the two sessions, as the subjects became more familiar with the experimental environment and the nociceptive stimuli in T2 session than in T1 session. It is be possible that with the adaptation to the experiment, the subjects are less anxious and less fearful to nociceptive laser stimuli, as such the attentional level to nociceptive stimuli and the stimulated site might be decreased in T2 session as compared with T1 session.

In line with the decreased brain responses in the S1, baseline spontaneous brain activity in the right S1 was also decreased from T1 session to T2 session (Fig. 2a). It is well recognized that the S1 is highly associated with the sensory-discriminative aspects of pain^30^, including the localization and discrimination of stimulus intensity, and its activation is easily modulated by several psychological factors (e.g., anticipation and attention)^31–33^. Mennes and colleagues demonstrated that a region's task-evoked BOLD responses could be predicted by that region's spontaneous brain activity^34^. For this reason, the changes in baseline spontaneous brain activity in the S1 could influence the laser-evoked brain responses in this region, which may lead to the decrease in sensory-discriminative processing of pain and the decrease in pain perception from T1 session to T2 session. On the other hand, S1 activation is highly modulated by cognitive factors that alter pain perception, including attention and previous experience^31^. With the adaption to the experimental environment and nociceptive stimuli, the focus of attention on nociceptive stimuli and the stimulated site was likely reduced in T2 session as compared with T1 session, which would contribute to the decrease of S1 activation and the decline of the self-report of pain perception.

Furthermore, resting-state functional connectivity between right S1 and bilateral dorsolateral prefrontal cortex was lower in T2 session as compared with T1 session (Fig. 2b). Previous studies demonstrated that the dorsolateral prefrontal cortex is generally related to the cognitive and attentional processing of painful stimuli^35,36^. Thus, the decreased functional connectivity between right S1 and the dorsolateral prefrontal cortex is likely related to the changed attentional state to nociceptive stimuli from T1 session to T2 session. Altogether, the decreased baseline functional connectivity might influence both laser-evoked pain perception and brain responses by allocating fewer attention resources to nociceptive stimuli.

Pain perception is a multidimensional experience that encompasses sensory-discriminative, affective-motivational, and cognitive-evaluative components^37^. As a consequence, pain perception and the associated brain responses are considerably variable and have fair-to-moderate reliability across experimental sessions. The variability between different experiment sessions of laser-evoked pain perception and brain responses could be related to the changes of baseline mental state that could be easily influenced by several physiological factors (e.g., anxiety level, the fearful level to the stimuli, and the attention level). Hence, assessing the possible effects of psychological state on pain perception and brain responses and monitoring the baseline brain activity are highly needed when designing experiments for laboratory studies and evaluating pain abnormalities in clinical practice. For instance, the across-session variability should be taken into consideration when (1) examining the test–retest reliability of pain perception from multiple experimental sessions; (2) assessing the habituation effect of pain using repeated painful stimulation over several days; and (3) evaluating the long-term analgesic effect of treatment strategies in clinical practice or basic research. Besides, evidence showed that structural MRI features (e.g., gray matter intensity/volume, cortical thickness, subcortical volume) are highly correlated with pain perception in both healthy subjects^38,39^ and chronic pain patients^40–42^. Therefore, investigating the relationship between structural MRI features and the test–retest reliability of pain perception or brain responses in future work would provide new insights into the neural mechanism of test–retest reliability of pain.

## Methods

The experiment was approved by the ethical standards of the Institutional Review Board of the Institute of Psychology, Chinese Academy of Sciences. All procedures were performed in accordance with the Declaration of Helsinki. Written informed consent was obtained from each subject prior to data collection. Subjects were screened for DSM-IV axis I and II disorders using the Structured Clinical Interview for DSM-IV, and were excluded if they had any contraindications (e.g., brain lesions, non-removable metallic implants, claustrophobia, etc.) for MRI scanning, history of peripheral and neurological disorders, chronic pain disorders, or if they consumed medications and reported a history of alcoholism or drug abuse.

### Pain sensitivity measurement

For each subject, pain sensitivity was assessed using the Pain Sensitivity Questionnaire^43^, and pain threshold was evaluated using laser stimuli. Radiant-heat laser stimuli were generated by an infrared neodymium yttrium aluminum perovskite (Nd:YAP) laser with a wavelength of 1.34 μm and a pulse duration of 4 ms (Electronical Engineering, Italy)^44^. At this wavelength and pulse duration, laser stimuli activate directly nociceptive terminals in the most superficial skin layers in a synchronized fashion^45^. A He–Ne laser pointed to the area to be stimulated. The laser pulse was transmitted via an optic fiber and focused by lenses to a spot with a diameter of ~ 7 mm (~ 38 mm^2^). Laser pulses were delivered to a squared area (4 × 4 cm^2^) on the dorsum of subjects’ left hand (non-dominant hand). To prevent fatigue or sensitization of the nociceptors, the laser beam target was manually shifted by at least 1 cm in a random direction after each stimulus^46–48^. The method of limits was adopted for each subject to determine the pain threshold in each session: starting from 1.5 J, the laser energy was increased in steps of 0.25 J until a rating of 4 out of 10 (i.e., the energy at which the subjects start to feel pain) was obtained on a numerical rating scale (NRS) ranging from 0 (no pain) to 10 (the worst pain imaginable)^44^. Prior to the pain threshold measurement, the skin temperature of the left hand was recorded for each subject to monitor the possible effect of temperature on pain perception.

### Experiment design

**Figure 3:**
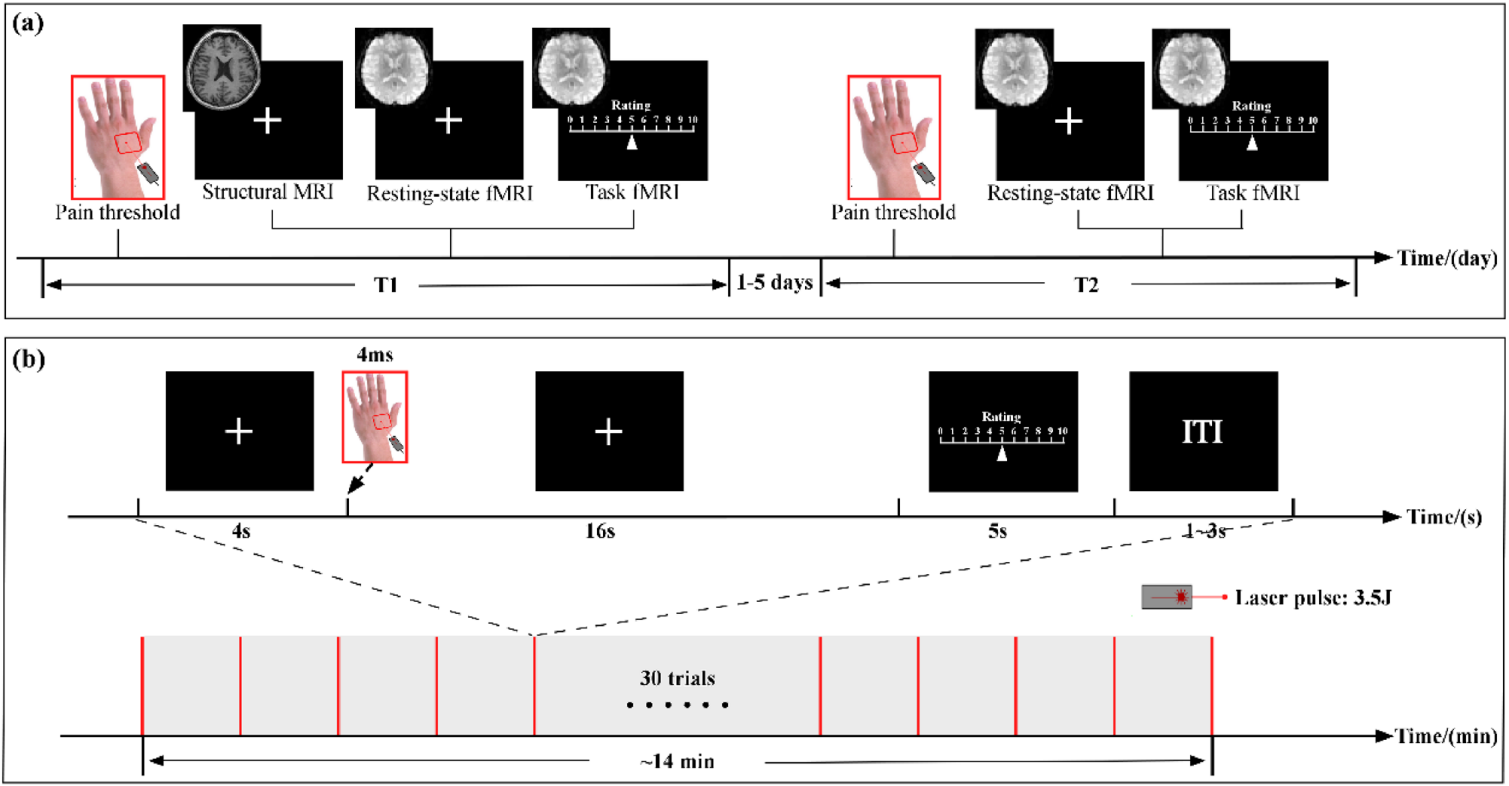
Experiment design and nociceptive stimulation. (**a**) Experimental design. (**b**) Task fMRI scan contained a single block of 30 trials with transient nociceptive stimuli. Each trial started with a 4-s fixation of the white cross-centered on the screen and followed by the delivery of a nociceptive stimulus. A visual cue presented 16 s after the nociceptive stimulus prompted the subjects to rate the perceived intensity within 5 s on the 0–10 NRS. The inter-trial interval varied randomly between 1 and 3 s. NRS, numerical rating scale. The MRI images were created by using the MRIcron software (v1.0.20190902, https://www.nitrc.org/projects/mricron).

An experiment with a within-subject repeated-measures design was performed (Fig. 3a). Each subject participated in two sessions (session 1: T1; session 2: T2) separated by 1–5 days (1.82 ± 1.12 days). In both T1 and T2 sessions, resting-state and task fMRI data were collected for each subject. To monitor subjects’ mood state, depression was evaluated by Beck-Depression Inventory (BDI)^49^ in T1 session, and state and trait anxiety was evaluated by State-Trait Anxiety Inventory (STAI)^50^ in both sessions.

During the task fMRI scan, 30 laser pulses at fixed stimulus energy of 3.5 J were delivered to the dorsum of the left hand to elicit a painful pinprick sensation for each subject (Fig. 3b). Inter-stimulus interval varied randomly between 26 and 28 s with a rectangular distribution. Each trial started with a 4-s white cross centered on the screen and followed by the laser stimulus (duration = 4 ms). A visual cue presented 16 s after the laser stimulus prompted the subjects to rate the intensity of pain perception elicited by the laser stimulus within 5 s on the same NRS. The following trial started in 1–3 s, and the duration of the whole task fMRI scan was ~ 14 min.

### MRI data acquisition

The MRI data were collected using a 3.0-Telsa Magnetic Resonance Imaging (MRI) system with a standard birdcage head coil (Discovery MR 750; General Electric Healthcare; Milwaukee; Wisc.) at the MRI Research Center, Institute of Psychology, Chinese Academy of Sciences. T1-weighted structural image was acquired using the gradient echo (3D SPGR) sequence (1 mm^3^ isotropic voxel, field of view (FOV) = 256 × 256 mm^2^). An echo-planar-imaging sequence (repetition time = 2000 ms; echo time = 30 ms; flip angle = 90°; FOV = 64 × 64 mm^2^; data matrix = 64 × 64; in-plane resolution = 3 × 3 mm^2^; slice thickness = 3.5 mm; slice spacing = 0.5 mm) was used to acquire the resting-state functional data (300 volumes for resting-state fMRI session), and then the task fMRI data (409 volumes for task fMRI session). During the resting-state fMRI data acquisition, the screen presented a black fixation ‘ + ’ in the center of the gray background. The patients were instructed to remain still with their eyes open, not to fall asleep, and not to think about anything in particular.

### Task fMRI data processing

Task fMRI data were analyzed using the 12th edition of Statistical Parametric Mapping (SPM12, Welcome Department of Cognitive Neurology, London, UK, http://www.fil.ion.ucl.ac.uk/spm). The preprocessing analysis included the following procedures^3^: discarding the first three volumes, slice timing correction, realignment and normalization to the EPI template with the resampling voxel size of 3 × 3 × 3 mm^3^, spatial smoothing using a Gaussian kernel of 5 mm full width at half maximum (FWHM), and temporal filtering using a high pass filter and cut-off at 128 s.

Statistical analysis of individual preprocessed imaging data was performed with first-level fixed-effects analyses using a general linear model. The regressor (nociceptive stimuli) was convolved with the canonical hemodynamic response function plus its temporal derivatives with realignment parameters (three translations and three rotations) included as regressors of no interest. For each subject, the contrast image of nociceptive stimuli was extracted to examine laser evoked BOLD responses within each group using a one-sample t-test. The resulted t-map of each session (T1 or T2) was thresholded at voxel-wise *p* < 0.005 and cluster-corrected at FWE of *p* < 0.05 at the whole-brain level. The differences in brain responses to nociceptive stimuli between sessions were assessed using a paired-sample t-test (voxel-wise *p* < 0.005 and cluster-corrected at FWE of *p* < 0.05).

### Test–retest reliability

Test–retest reliability is the extent to which a dependent variable is consistent and free from error across sessions^51^. The ICC is a widely-used measure of test–retest reliability. The ICCs and their 95% confidence intervals were calculated for pain threshold, pain rating, and laser-evoked BOLD responses between sessions 1 and 2 to evaluate their test–retest reliability using SPSS for Windows (Statistical Package for Social Sciences, Release 18.0, SPSS Inc., Chicago, IL, USA). In detail, ICCs of the absolute agreement were calculated using a two-way mixed model that provided a measure of consistency through a ratio of between-subject variance to the total variance^52^. For laser-evoked BOLD responses, mean BOLD data (defined in terms of parameter estimates) of clusters showing significant differences between the two sessions were extracted to calculate the ICCs. Besides, to assess the stability of brain activations in all regions of the “Pain Matrix”^18–21^, a series of ROIs, including the S1 and S2, thalamus, the insula, and the ACC, were selected. The ROIs of S1, S2, thalamus, and ACC were defined from the Harvard Oxford cortical and subcortical structural atlas^53,54^, which are population-based probability atlas in MNI 152 standard space. Four ROIs of the left and right anterior and posterior insula were defined as in our previous study^55,56^ using the analytic scripts downloaded via http://fcon_1000.projects.nitrc.org^57^. For each ROI, the mean BOLD response across all voxels within the ROI, defined in terms of parameter estimates, was extracted from each subject and each session. The degree of reliability of each value was then classified based on the conservative criteria described by Shrout^58^: virtually no reliability (0.00–0.10), slight-reliability (0.11–0.40), fair-reliability (0.41–0.60), moderate-reliability (0.61–0.80), and substantial reliability (0.81–1.00). Bonferroni procedures was performed to correct for multiple comparisons (*p* < 0.025 for pain perception analysis and *p* < 0.05/15 for brain response analysis).

### Resting-state fMRI data processing

Resting-state fMRI data were analyzed using Data Processing & Analysis for (Resting-State) Brain Imaging (DPABI) software (DPABI_V5.0_201001, http://rfmri.org/dpabi)^59^. The first ten volumes were discarded for signal equilibrium and to allow the participants’ adaptation to the scanning noise. Images were slice timing corrected and realigned. Structural images were segment into grey matter, white matter, and cerebrospinal fluid. White matter signal, cerebrospinal fluid signal, and head motion parameters were regressed as nuisance regressors from the corrected functional images. The processed functional data were then normalized to EPI template (resampling voxel size = 3 × 3 × 3 mm^3^), spatially smoothed using a Gaussian kernel of 5 mm FWHM, and detrended to remove the drifts and trends in the fMRI data.

Being defined as the ratio of the power spectrum of low-frequency fluctuations (0.01–0.1 Hz) to that of the entire frequency range (0–0.25 Hz), the fALFF were used to detect the intensity of regional spontaneous brain activity with high sensitivity and specificity^60–63^. To assess the spontaneous brain activity differences between the two sessions, fALFF was extracted and compared using a paired-sample t-test. Statistical maps were thresholded using the GRF theory correction procedure^64,65^, as implemented in DPABI^59^. The significance level was set at *p* < 0.05 corrected for multiple comparisons (paired-sample t-test, voxel z value > 2.3, cluster significance: *p* < 0.05, GRF corrected, two-tailed).

Brain regions showed significant differences of fALFF between the two sessions were selected as ROIs for the functional connectivity analysis of resting-state fMRI data. For each ROI, the time series of spontaneous brain activity, which were averaged across all voxels within the ROI, were extracted in each subject, and individual-level correlation maps of all voxels that were positively or negatively correlated with the averaged ROI time series were produced^55,56^. The resulting correlation maps were converted to Z-value maps using Fisher’s r-to-z transformation. A paired-sample t-test was performed to investigate the differences of resting-state functional connectivity for each ROI between the two sessions. Statistical maps were thresholded using the GRF theory correction procedure^59,64,65^, in which the significance level was set at *p* < 0.05 corrected for multiple comparisons (voxel z value > 2.3, cluster significance: *p* < 0.05, GRF corrected, two-tailed).
